# Zoonotic Marine Nematode Infection of Fish Products in Landlocked Country, Slovakia

**DOI:** 10.3201/eid2912.230674

**Published:** 2023-12

**Authors:** Mikuláš Oros, Daniel Barčák, Daniela Antolová, Miroslava Fecková, Tomáš Scholz

**Affiliations:** Institute of Parasitology, Slovak Academy of Sciences, Košice, Slovakia (M. Oros, D. Barčák, D. Antolová, M. Fecková);; Institute of Parasitology, Biology Centre of the Czech Academy of Sciences, České Budějovice, Czech Republic (T. Scholz);; University of South Bohemia Faculty of Science, České Budějovice (T. Scholz)

**Keywords:** anisakiasis, Anisakis, food safety, zoonoses, parasites, marine nematodes, Atlantic herring, fish products, Slovakia

## Abstract

Fish products in Slovakia have been heavily infected with *Anisakis* spp. larvae, which causes human anisakiasis. We found larvae in all tested samples of frozen Atlantic herring. Anisakid allergen t-Ani s7 testing revealed 2 positive cases in humans, signaling need for health authorities to closely monitor zoonotic marine parasites, even in inland areas.

Food safety is an unquestioned global public health imperative. Strict controls on food before release to markets are intended to prevent disease caused by agents of infectious diseases, including parasitoses such as human anisakiasis. Anisakiasis, an emerging zoonosis, is caused mainly by marine nematodes of the genus *Anisakis*. They mature in dolphins and whales, but their third-stage larvae, which reside in the flesh, gonads, and body cavities of marine fish, can infect humans who consume raw or undercooked fish (*1*). Most human cases are reported in Japan, Spain, and South Korea (*1*). 

Zoonotic diseases caused by marine parasites have been largely confined to coastal regions, but surveillance by health authorities in landlocked countries is lacking. However, global trade and the increasing popularity of raw fish consumption have contributed to emergence of that disease. We provide data on extensive infection of fish products with *Anisakis* larvae in Slovakia, a landlocked country in central Europe. We also report seropositive cases in a group of volunteers regularly eating fish products. We conducted this study in accordance with ethics standards in the 2013 revision of the Declaration of Helsinki of 1975. It was approved by the ethics committee of the Institute of Parasitology, Slovak Academy of Sciences (EC/01/2018; December 14, 2018). 

We examined 100 frozen Atlantic herring (*Clupea harengus*) provided by a fish product supplier and 18 packages of ready-to-eat pickled herring from local supermarkets for anisakid larvae. We found 4,163 larvae in frozen Atlantic herring at an intensity of infection of 2–368 (mean 42) larvae/fish (Figure, panels A–C, E–H). Although we found most larvae in the abdominal cavity, we also found them in the muscles of 1/3 and the gonads of 1/5 of fish we examined. Although all larvae were dead, even dead parasites or their residues in contaminated fish products can cause allergic reaction in sensitized persons (*2*,*3*). In addition, we found anisakids in 1/3 of ready-to-eat pickled herring (1–9 larvae/fish) (Figure, panel D). 

**Figure:**
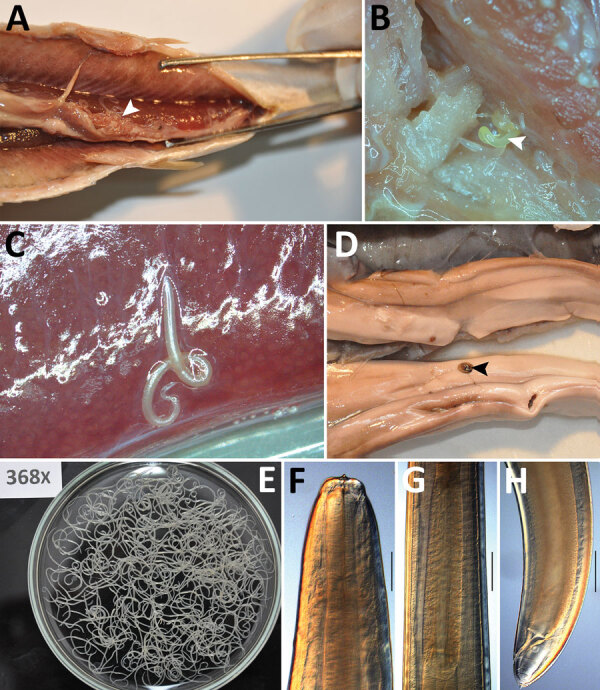
Anisakid larvae found in frozen (A–C, E–H) and ready-to-eat (D) Atlantic herrings (*Clupea harengus*), Slovakia. A) Cluster of larvae on the viscera (arrowhead). B) Larva in dorsal muscle (arrowhead). C) Larva partly embedded in the hard roe. D) Larva on the surface of the soft roe (arrowhead). E) All larvae isolated from single fish. Original magnification as indicated. F–H) Light microscope photographs of head part (F), ventriculus (G), and tail part (H) of an *Anisakis simplex* sensu stricto nematode. Scale bars indicate 200 µm.

We morphologically identified larvae as *Anisakis* spp. and used several larvae for genotyping based on the ≈800 bp–long internal transcribed spacer region. Sequences of all isolates were identical to those of *A. simplex* sensu stricto (Appendix), considered the predominant agent of human anisakiasis (*4*). 

We used a Trisakis 170, *A. simplex* IgE-ELISA kit (Laboratorio de Parasitología, Facultad de Farmacia, Universidad de Santiago de Compostela, Santiago de Compostela, Spain) to test IgE sensitization to *Anisakis* spp. in a human population. The kit detects IgE to the recombinant allergens r-Ani s 1 and t-Ani s 7 in human serum, which we collected in 2020 from 91 volunteers who regularly ate fish products. The allergen tests are highly specific (up to 100%), as deduced also from primary amino acid sequences of both allergens that have no significant homologies with other allergens to which humans are known to be sensitized. Sensitivity reaches 61.1% for Ani s 1 and 93.9% for Ani s 7; many researchers consider serum truly positive only when testing positive to Ani s 1 or Ani s 7 (*5*,*6*). Moreover, Ani s 7 and, probably, Ani s 1 are excretory-secretory allergens that are recognized by the host immune system only in the course of an *Anisakis* infection (5). Of 91 serum samples collected in 2020, sensitization to *Anisakis* allergen t-Ani s 7 was detected in 2 (2.2%) samples. The positive case-patients, both women, had no clinical symptoms of sensitization to *Anisakis* but had experienced allergy symptoms on several occasions in the past. 

Any report of sensitization to *Anisakis* in Slovakia is unusual in that it is a landlocked country. Given the low level of raw fish consumption in Slovakia, the 2.2% rate of detected positivity was relatively high. In a similar study in Norway, 0/993 blood donors and 1/414 (0.2%) patients with high IgE levels tested positive for anisakiasis (*6*). In a study in Croatia in which the same ELISA method was used as in our study, 3.5% positivity was found in persons living on islands, but only 1.5% in persons in urban areas on the coast (*7*). A 15.4% positivity rate was detected in an adult population in Spain (*8*), whereas seroprevalence was only 0.4% in blood donors in Galicia, in northwestern Spain (*9*). In the future, IgE for Ani s 1, Ani s 4, Ani s 5, and Anis s 9, heat-resistant allergens that cause most clinical episodes of the allergic form of anisakiasis, should be evaluated.

Monitoring fish products intended for human consumption for parasites currently receives insufficient attention. Although all parasites we found were dead, frequent presence of *Anisakis* spp. in herring poses a potential risk to sensitive persons who might suffer a hyperallergic reaction. In addition, some *Anisakis* larvae can survive freezing (*10*), so risk of infection remains even in fish products frozen for a short time. In conclusion, the results of our study signal the need for health authorities to closely monitor marine parasites with zoonotic potential, even in inland areas. 

Appendix Additional information about study of zoonotic marine nematodes in fish products in Slovakia.
