# Supplementary material for: Zoonotic Marine Nematode Infection of Fish Products in Landlocked Country, Slovakia
Source: Emerg Infect Dis. 2023 Dec;29(12):2578–80. doi: 10.3201/eid2912.230674 (PMC10683800; doi:10.3201/eid2912.230674)
Supplement: Appendix — Additional information about study of zoonotic marine nematodes in fish products in Slovakia. [file 23-0674-Techapp-s1.pdf]

Article DOI: https://doi.org/10.3201/eid2912.230674

EID cannot ensure accessibility for supplementary materials supplied by authors. Readers who have difficulty accessing supplementary content should contact the authors for assistance.

Zoonotic Marine Nematode Infection of Fish Products in Landlocked Country, Slovakia

Appendix

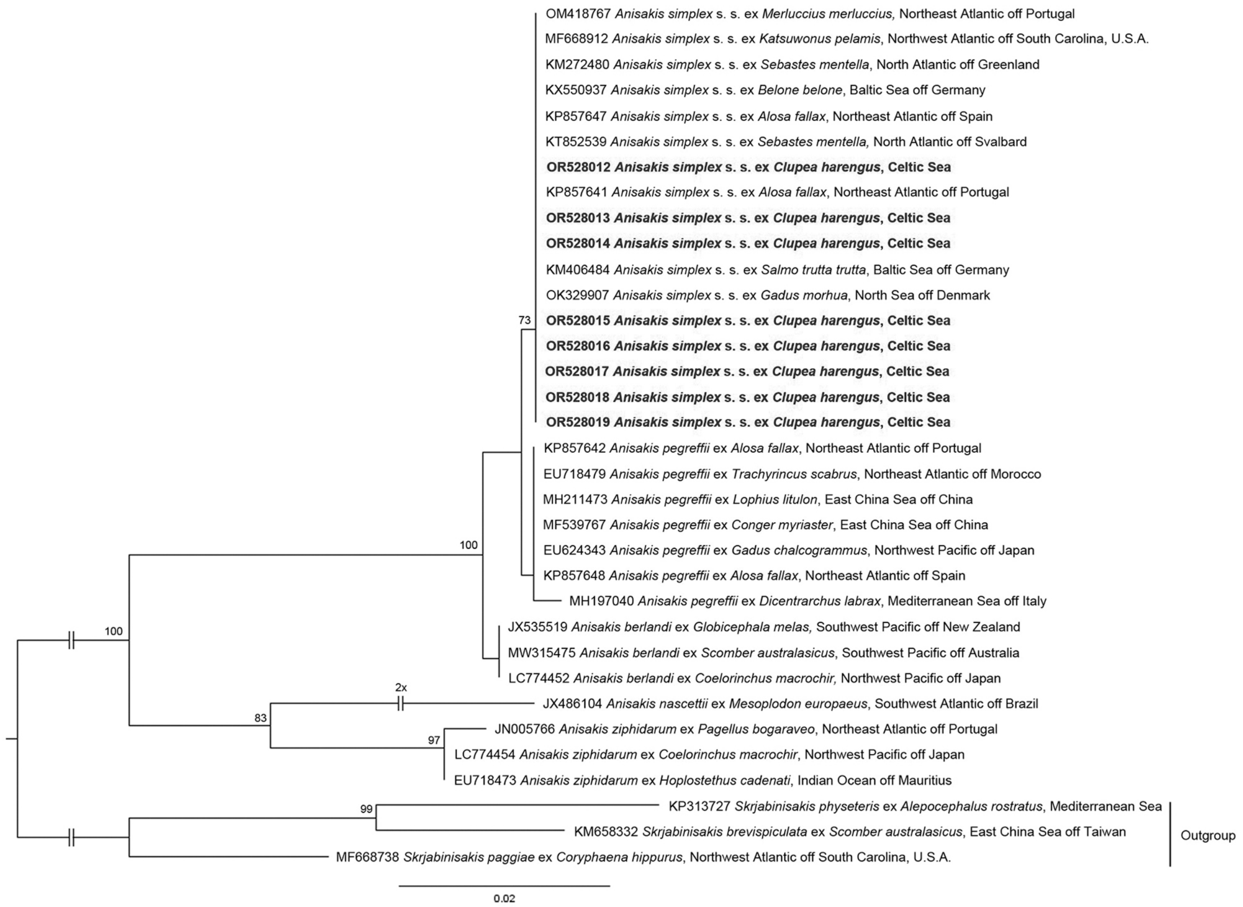

Appendix Figure. Phylogeny inferred from ITS rDNA dataset, maximum likelihood estimation by ultrafast bootstrap approximation with 1,000 replicates in IQ-TREE, version 2.0.5. Bootstrap supports over 70 shown. Newly generated sequences of *A. simplex* from fish products in Slovakia are bolded. Scale bar indicates number of substitutions per site.
